# Identification of Sinapic Acid Derivatives from Petit Vert Leaves and Their Effects on Glucose Uptake in C2C12 Murine Myoblasts

**DOI:** 10.3390/biom14101246

**Published:** 2024-10-01

**Authors:** Shizuo Yamada, Tsutomu Warashina, Osamu Shirota, Yoshihisa Kato, Toshiyuki Fukuda

**Affiliations:** 1 Center for Pharma-Food Research (CPFR), Graduate School of Pharmaceutical Sciences, Shizuoka 422-8526, Japan; 2 School of Food Nutritional Sciences, University of Shizuoka, Shizuoka 422-8526, Japan; warashin@u-shizuoka-ken.ac.jp; 3 Kagawa School of Pharmaceutical Sciences, Tokushima Bunri University, Sanuki 769-2193, Japan; oshirota@kph.bunri-u.ac.jp (O.S.); kato@kph.bunri-u.ac.jp (Y.K.); 4 Shizuoka Shin-Food Development Corp., Shizuoka 422-8064, Japan; fukudat.ssfd@gmail.com

**Keywords:** Petit vert leaves, sinapic acid, glucose uptake

## Abstract

Petit vert (scientific name: *Brassica oleracea* var. *gemmifera* DC. × *Brassica oleracea* var. *acephala* DC.) is a new variety of vegetable created by crossbreeding kale and brussel sprouts *(Brassica oleracea* species). The present study aimed to identify biologically active compounds in extracts of the outer leaves of Petit vert by purification and to examine their biological activities. The dried and powdered outer leaves of Petit vert were extracted, fractionated, and purified to isolate active compounds. Mass spectrometry (MS) was used to identify the compounds, and nuclear magnetic resonance (NMR) spectroscopy was performed to elucidate their structures. The compounds isolated from Petit vert leaves were glycosides that contained kaempferol, quercetin (flavonol), or sinapic acid (phenylpropanoid). Glucose uptake in cultured C2C12 murine myoblasts in the absence of insulin was significantly increased by these compounds, kaempferol, sinapic acid, and ferulic acid, while uptake in the presence of insulin was also significantly increased by compounds **3** and **4**, kaempferol, and sinapic acid. The effect was not necessarily concentration-dependent, and some agents decreased the glucose uptake at higher concentrations. The present study reports for the first time the isolation of five compounds containing sinapic acid from the outer leaves of Petit vert and their stimulation of glucose uptake in cultured C2C12 murine myoblasts. The results obtained herein suggest the potential of these compounds to effectively attenuate hyperglycemia and maintain muscle strength by promoting glucose metabolism in muscle cells.

## 1. Introduction

Polyphenols are phenolic compounds that are present in plants and play a role in defense against harmful oxidative damage. Among polyphenol compounds, hydroxycinnamic acid and its derivatives are well-known chemical groups that have important biological functions, such as anti-inflammatory and antioxidant activities [1]. The beneficial effects of these compounds as preventive or therapeutic agents have previously been shown in various diseases, such as inflammatory damage and cancer [2,3]. Among hydroxycinnamic acids, sinapic acid [(2*E*)-3-(4-hydroxy-3,5-dimethoxyphenyl)prop-2-enoic acid], a natural herb phenolic acid compound, is present in herbs, such as oranges, grapefruits, cranberries, rapeseeds, and mustard seeds [4]. Sinapic acid was previously shown to prevent scopolamine-induced cognitive impairments in memory in a rat model [5,6]. It also exhibited anti-inflammatory activity in carbon tetrachloride-induced acute liver damage and significantly decreased proinflammatory cytokine levels [7,8]. Furthermore, sinapic acid attenuated and prevented neuroinflammation, cholinergic deficits, and oxidative stress [9]. 

**Figure 1:**
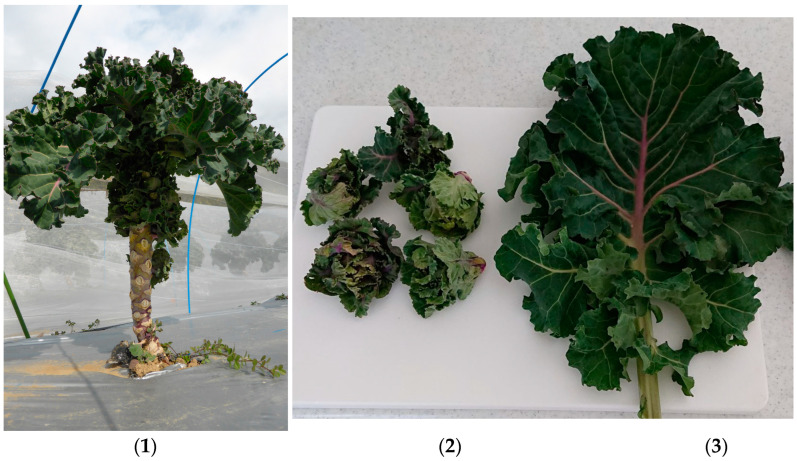
Petit vert (scientific name: *Brassica oleracea var. gemmifera* DC. × *Brassica oleracea* var. *acephala* DC.). (**1**) Petit vert. (**2**) Side shoots. (**3**) Leaf.

Petit vert (scientific name: *Brassica oleracea* var. *gemmifera* DC. × *Brassica oleracea* var. *acephala* DC.) (Figure 1) is a new variety of vegetable created by crossbreeding kale and brussel sprouts *(Brassica oleracea* species). Its anti-obesity effects were previously demonstrated in mice fed a high-fat diet containing 5% freeze-dried Petit vert [10]; however, the composition and identity of the active compounds present in Petit vert remain unclear. Since only the leafy side shoots of Petit vert are harvested, the outer leaves, which are of high nutritional value, are typically discarded because they contain rough fibers. Therefore, the present study aimed to identify biologically active compounds in extracts of the outer leaves of Petit vert by purification and to examine their biological activities. Dried and powdered outer leaves of Petit vert were extracted, fractionated, and purified to isolate active compounds. Nuclear magnetic resonance (NMR) and mass (MS) spectrometry were performed to elucidate the structures of five compounds. The compounds isolated from Petit vert leaves were glycosides containing either kaempferol, quercetin (flavonol), or sinapinic acid (phenylpropanoid).

## 2. Materials and Methods

### 2.1. Materials

Petit vert (*B. oleracea* var. *gemmifera* DC. × *B. oleracea* var. *acephala* DC.) is an agricultural product. It was cultivated by seedlings and purchased from Masuda Seed Farm Col. Ltd. (Shizuoka, Japan). Test compounds (**1**–**5**) were isolated from Petit vert. Quercetin (**6**), kaempferol (**7**), sinapic acid (**8**), ferulic acid (**9**), and caffeic acid (**10**) were purchased from Merck KGaA (Darmstadt, Germany). Penicillin-streptomycin solution, a 0.25% trypsin/EDTA solution, cell count reagent SF (WST-8), and Dulbecco’s modified Eagle’s medium (DMEM) were purchased from Nacalai Tesque Inc. (Kyoto, Japan). Gelatin, type A and Gibco™ Insulin, human recombinant, zinc solution were purchased from MP Biomedicals (Irvine, CA, USA) and Thermo Fisher Scientific (Waltham, MA, USA), respectively. Fetal bovine serum (FBS) was obtained from Nichirei Biosciences Inc. (Tokyo, Japan). All other chemicals used were of the highest purity commercially available. All solvents were of high-performance liquid chromatography (HPLC) grade, and water was of Milli-Q quality.

### 2.2. Extraction and Isolation

Petit vert is usually harvested from February to March in Japan. The outer leaves were harvested at the same time. Petit vert leaves were dried at 55 °C for 7 h and ground to a powder in a domestic blender. Polyphenols were extracted from 400 g of dried Petit vert leaf powder with 6 L of methanol under reflux conditions (around 70 °C) for 2 h before filtering. The filtrate was evaporated, and the extract was suspended in 1.3 L of 90% aqueous methanol. The suspension was washed three times with 1.3 L of *n*-hexane, and the extract was evaporated and suspended in 1.3 L of distilled water. The extract was washed three times with 1.3 L of ethyl acetate. The extract was again evaporated and then dissolved in 1 L of distilled water. The extract was charged on a porous polymer gel (DIAION HP-20, Mitsubishi Chemical Co., Chiyoda City, Japan) column and eluted with 3 L of distilled water, 3 L of 50% aqueous methanol, and 3 L of methanol. Extracts were evaporated to obtain the phenolic extract (103.7, 8.4, and 2.4 g, respectively). The 50% aqueous methanol eluate (7.6 g) was charged on a TOYOPEARL HW-40 column (TOSOH, Tokyo, Japan) and then eluted with 10% aqueous ethanol and 50% aqueous ethanol to obtain eight fractions (fractions A–H). Fraction C (705 mg) was purified by preparative HPLC (Inertsil ODS-3, i.d., 30 × 250 mm, PU714Mx2, GL Sciences Inc., Tokyo, Japan), and compounds **2**–**5** (**2**; 41.6 mg, **3**; 34.8 mg, **4**; 90.5 mg, **5**; 53.8 mg), which were of sufficient purity and quantity for complete chemical characterization, were isolated. The MeOH eluate (2.4 g) was charged on a TOYOPEARL HW-40 column (TOSOH, Tokyo, Japan) and then eluted with 50% aqueous ethanol and 85% aqueous ethanol to obtain six fractions (fractions A–F). Fraction B (785 mg) was purified by preparative HPLC (Inertsil ODS-3, i.d., 30 × 250 mm, GL Sciences Inc., Tokyo, Japan), and compound **1** (33.4 mg) was isolated.

### 2.3. Identification of Phenolic Compounds by HPLC

To isolate flavonoids and hydroxycinnamic acid derivatives, HPLC (CBM-20A, Shimadzu, Japan) consisting of a degasser, quaternary pump, thermostat, a UV detector set at 330 nm, and an injection valve was used. Extracts were analyzed on a Cosmosil 2.5 Cholester column (100 × 3 mm, 2.5 µm; Nacalai Tesque, Inc.) at a temperature of 40 °C using a gradient of (A) 0.1% trifluoroacetic acid in water and (B) acetonitrile. The following gradient with a flow rate of 0.75 mL/min was used: 6–11% B (0–20 min), 11–27% B (20–36 min), and 27% B isocratic (36–45 min).

### 2.4. Structural Elucidation of Phenolic Compounds by NMR and MS

The structures of compounds **1**–**5** were identified by NMR spectroscopy. ^1^H and ^13^C NMR spectra were analyzed on a JNM ECA-500 spectrometer using the Delta/NMR software system (JEOL, Tokyo, Japan) (500 MHz for ^1^H and 125 MHz for ^13^C) or a Bruker Avance 700 spectrometer using the MestReNova software system (Bruker, Billerica, MA, USA) (700 MHz for ^1^H and 175 MHz for ^13^C) in CD_3_OD or DMSO-*d*_6_. Chemical shifts are given in *δ* (ppm) using tetramethylsilane (TMS) as an internal standard. The 2D-NMR spectra were carried out with the same spectrometer. Inverse-detected heteronuclear correlations were measured using HSQC (optimized ^1^*J*_C-H_ = 140 Hz) and HMBC (optimized ^3^*J*_C-H_ = 8 Hz) pulse sequences with a pulse field gradient. The ROESY spectrum was recorded with a mixing time of 300 ms. High resolution (HR) electrospray ionization (ESI) MS data on compounds **2**–**5** were obtained on a Waters/Micromass Q-TOF micro MS using MassLynx software system (Waters, MA, USA) in the negative ion mode.

### 2.5. Cell Culture

C2C12 mouse myoblast cells were purchased from the American Type Culture Collection (Manassas, VA, USA). C2C12 cells form a myoblast cell line, which is a subclone (produced by H. Blau et al.) of the mouse myoblast cell established by D. Yaffe and O. Saxel. The study was conducted according to the guidelines of the Declaration of Helsinki (2013). The experiment was approved by the Committee for Research at the University of Shizuoka Research Ethical Board approved on 2 June 2019. Cells were cultured in DMEM with 25 mM glucose, 10% FBS, and 1% antibiotic solution containing penicillin and streptomycin at 37 °C in a humidified atmosphere of 5% CO_2_–95% air. Cells were plated in a 96-well microplate (100 µL well^−1^) at a density of 4 × 10^4^ cells well^−1^. Confluent myoblasts were differentiated to myotubes by a decrease in serum concentrations to 0.1% and the addition of 5 µg/mL insulin. Myotubes formed after 4 days of incubation and were used in subsequent experiments.

### 2.6. Cell Viability Assay

Cell viability assays were performed using the cell count reagent SF (WST-8). The WST-8 reagent solution (10 µL) was added to each well of a 96-well microplate containing 100 µL of a cell suspension in culture medium and the test compounds (**1**–**10**) at various concentrations (1, 10, and 100 µM). Plates were incubated at 37 °C for 24 h. Control wells (0.1% DMSO) containing the same volume of complete culture medium were included in each assay. After being cultured for 24 h, absorbance was measured at 450 nm using a microplate reader (Varioskan Flash, Thermo Fisher Scientific Waltham, MA, USA). Measurements were performed with triplicate dishes of three different experiments.

### 2.7. Assay of Glucose Uptake in C2C12 Mouse Myoblast Cells

The glucose uptake rate was measured using the Glucose Uptake-Glo^TM^ Assay kit (Promega Corporation, Madison, WI, USA) according to the manufacturer’s instructions. This assay measures glucose uptake in cells based on the detection of 2-deoxyglucose-6-phosphate. Test compounds (**1**–**10**) were added at various concentrations (1, 10, and 100 µM) to each well of a 96-well microplate containing cells in culture medium, and the plate was incubated at 37 °C for 24 h. Control wells (0.1% DMSO) containing the same volume of a complete culture medium were included in each assay. Before the assay on glucose uptake, cells were placed in a serum-free medium for 18 h. C2C12 mouse myoblast cells were then cultured in DMEM with 25 mM glucose and 10% FBS in the presence or absence of insulin and the test substances (1, 10, and 100 μM) for 1 h. Fluorescence intensity was measured using a fluorescence spectrofluorometer (Varioskan Flash, Thermo Fisher Scientific Waltham, MA, USA) and was used to calculate glucose in the cells.

### 2.8. Statistical Analysis

Data were statistically analyzed using Student’s *t*-test after an analysis of variance. Results are shown as the mean ± S.E. unless otherwise stated.

## 3. Results

### 3.1. Structural Elucidation of Phenolic Compounds by NMR

^13^C and ^1^H NMR spectroscopic data on compound **1** showed two characteristic anomeric proton and carbon signals for *β*-D-glucopyranosyl groups at δ_C_ 91.9, 103.1, δ_H_ 5.77 (1H, *d*, *J* = 8.0 Hz), 4.20 (1H, *d*, *J* = 8.0 Hz) and two *trans*-sinapoyl groups at δ_C_ 164.9, 148.0 × 2, 145.8, 138.4, 124.3, 113.5, 106.3 × 2, 56.1 × 2, δ_H_ 7.55 (1H, *d*, *J* = 16.0 Hz), 6.99 (1H, *s*) ×2, 6.49 (1H, *d*, *J* = 16.0 Hz), 3.77 (3H, s) × 2 and δ_C_ 165.6, 148.0 × 2, 147.3, 138.9, 124.0, 114.6, 106.6 × 2, 56.1 × 2, δ_H_ 7.53 (1H, *d*, *J* = 16.0 Hz), 6.96 (1H, *s*) × 2, 6.44 (1H, *d*, *J* = 16.0 Hz), and 3.78 (3H, *s*) × 2. Therefore, compound **1** consisted of two *β*-D-glucopyranosyl and two *trans*-sinapoyl groups. Consistent with NMR spectroscopic data on compound **1** in the literature, compound **1** was assigned as 1,2-disinapoylgentiobiose [11].

**Figure 2:**
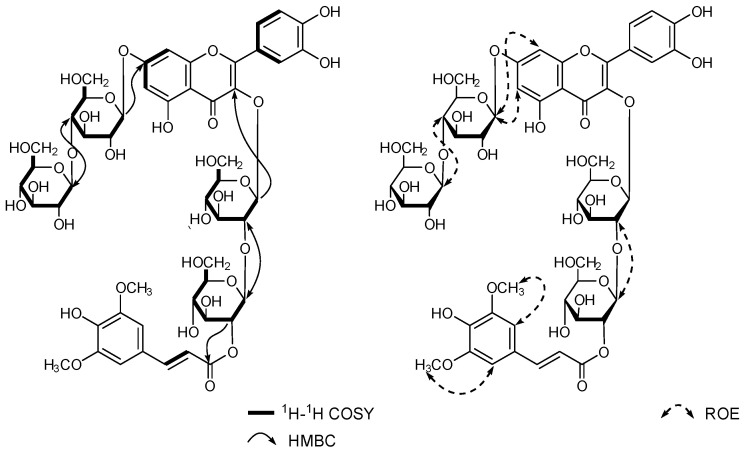
Observed key ^1^H-^1^H COSY, HMBC, and ROE correlations of compound **2**.

The molecular formula for new compound **2**, C_50_H_60_O_31_, was established based on HR-ESI-MS [*m*/*z*: 1155.3058 [M-H]^−^]. The aglycone of compound **2** was identified as quercetin based on observations of fifteen carbon signals, including twelve aromatic carbon signals (δ_C_ 164.1, 162.2, 157.3, 149.8, 145.9, 123.3 × 2, 117.2, 116.1, 107.5, 100.6, and 95.4), two olefin carbons (δ_C_ 157.5 and 135.0), and one carbonyl carbon (δ_C_ 179.1), and AM-type aromatic protons [δ_H_ 6.37 (1H, *d*, *J* = 2.0 Hz) and 6.34 (1H, *d*, *J* = 2.0 Hz)] derived from the A-ring and AMX-type aromatic proton signals [δ_H_ 7.50 (1H, *d*, *J* = 2.0 Hz) 7.45 (1H, *dd*, *J* = 8.5, 2.0 Hz) and 6.88 (1H, *d*, *J* = 8.5 Hz)] derived from the B-ring in ^13^C and ^1^H NMR spectroscopic data. Moreover, in the ^13^C and ^1^H NMR spectra of compound **2**, four anomeric carbon and proton signals of *β*-D-glucopyranosyl groups were observed at δ_C_ 96.9, 98.4, 101.2, 104.5, and δ_H_ 6.18 (1H, *d*, *J* = 8.0 Hz), 5.24 (1H, *d*, *J* = 8.0 Hz), 5.13 (1H, *d*, *J* = 8.0 Hz), and 4.48 (1H, *d*, *J* = 8.0 Hz) together with signals due to one *trans*-sinapoyl group [δ_C_ 168.6, 148.7 × 2, 146.6, 138.7, 125.9, 115.9, 105.5 × 2, 56.2 × 2 and δ_H_ 7.30 (1H, *d*, *J* = 16.0 Hz), 6.23 (1H, *s*) × 2, 6.09 (1H, *d*, *J* = 16.0 Hz), 3.59 (3H, *s*)×2]. These components of compound **2** were confirmed by alkaline and acid hydrolysis. ^13^C and ^1^H NMR signals were assigned (Table 1 and Table 2) based on the results of two-dimensional (2D)-NMR [^1^H-^1^H correlation spectroscopy (COSY), ^1^H-detected heteronuclear single-quantum correlation spectroscopy (HSQC), and ^1^H-detected hetero-nuclear multiple-bond connectivity (HMBC)] measurements. In addition, the HMBC experiment provided information on sugar and ester linkages. ^3^*J*_COCH_s were observed between C-3 of the aglycone (δ_C_ 135.0) and H-1 of *β*-D-glucopyranose (δ_H_ 6.18), C-2 of *β*-D-glucopyranose (δ_c_ 82.0), H-1′ of *β*-D-glucopyranose (δ_H_ 5.24), C-7 of the aglycone (*δ*_C_ 164.1), H-1″ of *β*-D-glucopyranose (δ_H_ 5.13), C-4″ of *β*-D-glucopyranose (δ_c_ 80.1), and H-1‴ of *β*-D-glucopyranose (δ_H_ 4.48). These sugar linkages were also indicated by the results of a rotating frame nuclear Overhauser effect correlation spectroscopy (ROESY) experiment (Figure 2). Moreover, the acylation shift of the H-2′ signal of *β*-D-glucopyranose (δ_H_ 4.93) suggested that the sinapoyl group was attached at C-2′ of *β*-D-glucopyranose, which was supported by the results of the HMBC experiment. Therefore, compound **2** was identified as quercetin 3-*O*-*β*-D-[2-*O*-3,5-dimethoxy-4-hydroxy-(*E*)-cinnamoyl]-glucopyranosyl-(1→2)-*β*-D-glucopyranoside-7-*O*-*β*-D-glucopyranosyl-(1→4)-*β*-D-glucopyranoside.

Based on HR-ESI-MS, compound **3** had the molecular formula C_44_H_50_O_26_, which was smaller by one glucosyl unit than that of compound **2** [*m*/*z*: 993.2518 [M-H]^−^]. ^13^C- and ^1^H-NMR spectroscopic data on the aglycone and 3-*O*-side chain were consistent with those of compound **2**. However, since the signals of only one *β*-D-glucopyranosyl group were observed in the 7-*O*-side chain, this chain consisted of one *β*-D-glucopyranose. Therefore, compound **3** was identified as quercetin 3-*O*-*β*-D-[2-*O*-3,5-dimethoxy-4-hydroxy-(*E*)-cinnamoyl]-glucopyranosyl-(1→2)-*β*-D-glucopyranoside-7-*O*-*β*-D-glucopyranoside [12,13].

**Figure 3:**
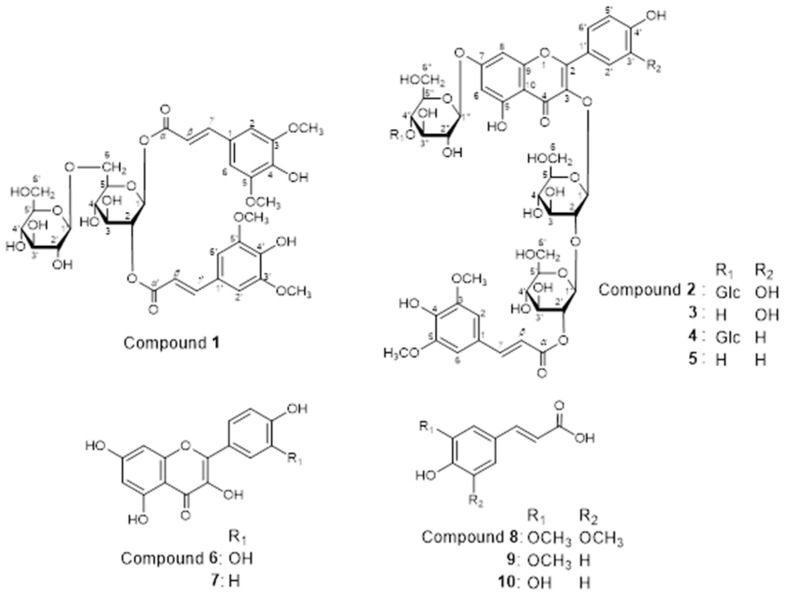
Structures of compounds **1**–**10**.

**Figure 4:**
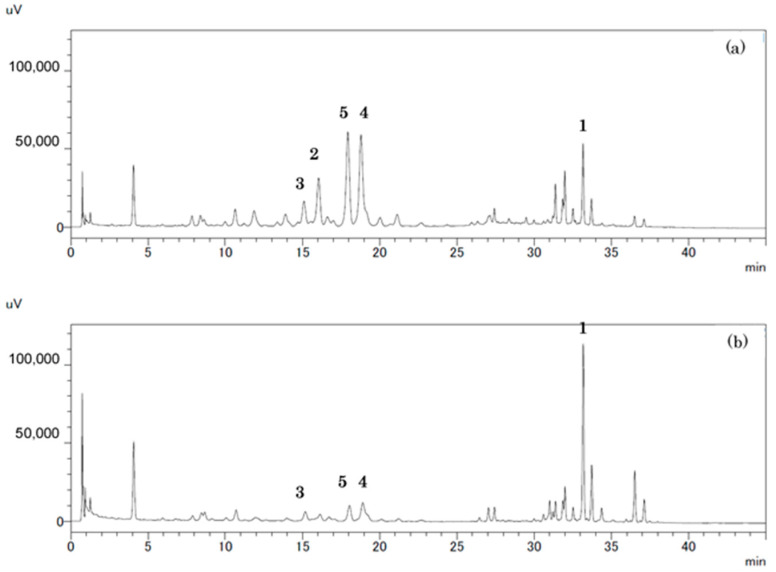
HPLC profiles of MeOH extracts from Petit vert leaf (**a**) and Petit vert side shoots (**b**). HPLC conditions [column: Cosmosil 2.5Cholester (2.5 µm), i.d. 3.0 × 100 mm (NACALAI TESQUE, INC.); mobile phase: A 0.1%TFA in water, B CH_3_CN, gradient, 0–20 min: 6–11% B linear, 20–36 min: 11–27% B linear, 36–45 min: 27% B; and flow rate: 0.75 mL/min; Detection: UV 330 nm; [Column temperature: 40 ℃].

Compounds **4** and **5** were assigned the molecular formulae C_50_H_60_O_30_ and C_44_H_50_O_25_, respectively, by HR-ESI-MS [compound **4**: *m*/*z*: 1139.3077 [M-H]^−^, compound **5**: *m*/*z*: 977.2565 [M-H]^−^]. The aglycones of compounds **4** and **5** were identified as kaempferol because AA′XX′-type aromatic proton signals derived from the B-ring were observed in each ^1^H NMR spectrum [*δ*_H_ 7.89 (1H, *d*, *J* = 8.5 Hz) × 2, 6.90 (1H, *d*, *J* = 8.5 Hz) × 2]. NMR spectroscopic data, due to the sugar and ester moieties of compounds **4** and **5**, were consistent with those of compounds **2** and **3**, respectively. Therefore, the structures of compounds **4** and **5** were elucidated, as shown in Figure 3 [12,13,14,15] (Chart S1). In addition to the sinapoyl group containing the compounds reported herein, some flavonoid glycosides containing *p*-coumaroyl, caffeoyl, or feruloyl groups in their molecules were obtained. Since Petit vert is an improved variety of *B. oleracea*, similarities in its composition with that of *B. oleracea* were observed [12,14,15]. The HPLC analysis of Petit vert leaves and side shoots demonstrated that compounds **2**–**5** with flavonols were more abundant in the leaves than in the side shoots. On the other hand, compound **1**, which contained only sinapic acid, was more abundant in the side shoots (Figure 4). 

### 3.2. Effects on Glucose Uptake in Cultured C2C12 Murine Myoblasts

We examined the effects of the following 10 compounds on glucose uptake in cultured C2C12 murine myoblasts: the 5 compounds isolated from the outer leaves of Petit vert (compounds **1**–**5**), quercetin, kaempferol (aglycone), and related acyl groups, including sinapic acid, ferulic acid, and caffeic acid. In a cytotoxicity assay, we measured the number of C2C12 cells after a 24 h incubation in a differentiation medium supplemented with the test substances (final concentrations: 1, 10, and 100 μM). As shown in Table 3, little significant change was observed in the viable cell rate (% of the control) in the presence of compounds **1**–**5**, quercetin, kaempferol, sinapic acid, ferulic acid, and caffeic acid at 1–100 µM, except for a significant decrease (23.4%, and 33.4%, respectively), which was noted for 100 µM quercetin and kaempferol.

As described in the “Materials and Methods” section, cultured C2C12 cells were treated with a differentiation medium containing the test compounds (1, 10, and 100 µM) in the presence or absence of insulin for 1 h, and glucose uptake levels in cells were measured. In comparison with the control, glucose uptake levels significantly increased in the absence of insulin when cells were cultured with compounds **1** (22.2–28.4%), **2** (30.9% at 1 µM), **3** (20.1–30.7%), **4** (27.4–45.5%), and **5** (14.9–16.8%) as well as with kaempferol (35.0–44.5%), sinapic acid (27.3–35.8%), and ferulic acid (16.2–20.5%), at 1, 10, and 100 µM (Table 4). A significant decrease was observed with compound **2**, quercetin, and kaempferol at 100 µM. Caffeic acid did not significantly affect glucose uptake. Furthermore, glucose uptake in the presence of insulin was significantly increased by compounds **3** (1 µM) and **4** (100 µM), kaempferol (1, 10 µM), and sinapic acid (1–100 µM) (Table 5). A significant decrease in glucose uptake was noted with compounds **3** (100 µM), **4** (1 µM), and **5** (100 µM), quercetin (1–100 µM), and kaempferol (100 µM). Ferulic acid and caffeic acid did not significantly affect glucose uptake.

## 4. Discussion

In the present study, a Petit vert extract was prepared by extracting its dried and powdered outer leaves with MeOH. The extract was subsequently analyzed by HPLC. We identified several peaks that appeared to correspond to polyphenols with absorption at approximately UV 330 nm. The dried and powdered outer leaves of Petit vert were extracted, fractionated, and purified to isolate five compounds (compounds **1**–**5**). NMR spectroscopy was performed for each of the components to elucidate their structures. Compounds **2**–**5** isolated from Petit vert leaves were glycosides that contained either kaempferol or quercetin with a sinapoyl group. Compound **1** was previously isolated from broccoli florets [11], compounds **2**, **3**, and **5** from kale leaves [12], and compounds **4** and **5** from cabbage leaves [14,15]. Compounds **1**–**5** all contained sinapic acid, which is an acyl group. According to the HPLC analysis of Petit vert leaves and side shoots, compounds **2**–**5** with flavonols were more abundant in the leaves than in the side shoots, while compound **1** containing only sinapic acid, was more abundant in the side shoots.

We investigated the effects of the following ten compounds on glucose uptake in cultured C2C12 murine myoblasts: five compounds were isolated from the outer leaves of Petit vert (compounds **1**–**5**), quercetin, kaempferol, and related acyl groups, including sinapic acid, ferulic acid, and caffeic acid. Glucose uptake in cultured C2C12 murine myoblasts in the absence of insulin was significantly increased by compounds **1**–**5**, kaempferol, sinapic acid, and ferulic acid (Table 4), while uptake in the presence of insulin was also significantly increased by compounds **3** and **4**, kaempferol, and sinapic acid (Table 5). The significant decrease detected in glucose uptake at higher concentrations of quercetin and kaempferol may have been due to a reduction in the viable cell rate (Table 3). The five compounds isolated from the outer leaves of petit vert, as well as quercetin, kaempferol, and sinapic acid, increased glucose uptake in cultured C2C12 murine myoblasts. These results indicate that the biological activities of the outer leaves of Petit vert, such as increases in glucose uptake, were the summed effects of the phenolic compounds present.

Phenolic acids and their derivatives compose one of the most common groups of phenolic compounds in plants. These molecules have emerged as high-value-added products because of their essential role in human nutrition and health [16,17]. Previous studies reported the effects of phenolic compounds, such as sinapic acid, related to those isolated from Petit vert leaves. For example, kaempferol was shown to exert anti-obesity effects because it increased insulin secretion in beta cells [18]. Sinapic acid has attracted increasing attention due to its numerous pharmacological effects [19]. It has been shown to exhibit antihyperglycemic [20], antioxidant [21], anti-inflammatory [22], anti-cancer [23], hepatoprotective [24], cardioprotective [25], renoprotective [26], neuroprotective [9], anti-diabetic [27], and anti-bacterial activities [28]. The compounds identified from the leaves of the Petit vert vegetable in the present study contain interesting compounds, such as sinapic acid, which have been previously reported to have ameliorative effects on various diseases at the preclinical level. Therefore, it is assumed that the consumption of this vegetable can contribute to human health in the future. 

## 5. Conclusions

The present study reports for the first time the isolation of five compounds containing sinapic acid from the outer leaves of Petit vert and their stimulation of glucose uptake in cultured C2C12 murine myoblasts. The results obtained herein suggest the potential of these compounds to effectively attenuate hyperglycemia and maintain muscle strength by promoting glucose metabolism in muscle cells. It is expected that the present study may become a trigger for the further elucidation of health benefits of Petit vert vegetables. 

## Figures and Tables

**Table 1:** ^13^C NMR spectroscopic data of flavonoid glycosides and a cinnamic acid derivative (δ/ppm; 175 or 125 MHz instruments).

Compound	1 ^a^	2 ^b^	3 ^b^	4 ^b^	5 ^b^
Aglycone moiety					
C-2	–	157.5	157.3	157.7	157.5
3	–	135.0	134.9	134.8	134.8
4	–	179.1	179.1	179.1	179.4
5	–	162.2	162.1	162.1	162.2
6	–	100.6	100.6	100.7	100.7
7	–	164.1	164.2	164.1	164.2
8	–	95.4	95.1	95.3	95.3
9	–	157.3	157.5	157.3	157.4
10	–	107.5	107.4	107.5	107.5
1′	–	123.3	123.3	122.9	123.0
2′	–	117.2	117.1	132.2	132.2
3′	–	149.8	149.7	116.2	116.1
4′	–	145.9	145.9	161.4	161.5
5′	–	116.1	116.1	116.2	116.1
6′	–	123.3	123.3	132.2	132.2
Suger moiety					
(3)-*O*-sugars					
C-1	91.9	96.9	96.9	97.0	97.0
2	73.5	82.0	82.0	82.0	82.1
3	73.8	75.1	75.1	75.1	75.1
4	70.0	71.4	71.3	71.5	71.5
5	76.7	78.3	78.3	78.2	78.3
6	67.8	62.2	62.2	62.2	62.3
C-1′	103.1	98.4	98.4	98.5	98.5
2′	72.6	75.0	75.0	75.0	75.0
3′	76.5	75.8	75.8	75.8	75.9
4′	70.0	71.2	71.1	71.2	71.2
5′	76.8	77.8	77.8	77.8	77.9
6′	61.0	62.2	62.2	62.2	62.2
7-*O*-sugars					
C-1″	–	101.2	101.4	101.2	101.5
2″	–	74.5	74.8	74.5	74.8
3″	–	76.0	77.7	76.0	77.7
4″	–	80.1	71.4	80.2	71.4
5″	–	76.6	78.1	76.6	78.2
6″	–	61.6	62.5	61.7	62.5
C-1‴	–	104.5	–	104.5	–
2‴	–	74.9	–	74.9	–
3‴	–	77.8	–	77.8	–
4‴	–	71.3	–	71.3	–
5‴	–	78.1	–	78.0	–
6‴	–	62.4	–	62.4	–
Ester moiety					
C-*α*	164.9	168.6	168.6	168.4	168.4
*β*	113.5	115.9	115.9	116.0	116.0
*γ*	145.8	146.6	146.6	146.5	146.5
1	124.3	125.9	125.9	126.0	126.0
2	106.3	105.5	105.5	105.6	105.6
3	148.0	148.7	148.7	148.7	148.8
4	138.4	138.7	138.7	138.7	138.8
5	148.0	148.7	148.7	148.7	148.8
6	106.3	105.5	105.5	105.6	105.6
OMe	56.1 × 2	56.2 × 2	56.2 × 2	56.3 × 2	56.3 × 2
C-*α*′	165.6	–	–	–	–
*β*′	114.6	–	–	–	–
*γ*′	147.3	–	–	–	–
1′	124.0	–	–	–	–
2′	106.6	–	–	–	–
3′	148.0	–	–	–	–
4′	138.9	–	–	–	–
5′	148.0	–	–	–	–
6′	106.6	–	–	–	–
OMe	56.1 × 2	–	–	–	–

^a^: Measured in DMSO-*d*_6_. ^b^: Measured in MeOH-*d*_4_.

**Table 2:** ^1^H NMR spectroscopic data of flavonoid glycosides and a cinnamic acid derivative (δ/ppm; 700 or 500 MHz instruments).

Compound	1 ^a^	2 ^b^	3 ^b^	4 ^b^	5 ^b^
Aglycone moiety					
H-6	–	6.37 (*d*, 2.0)	6.37 (*d*, 2.0)	6.38 (*s*)	6.38 (*d*, 2.0)
8	–	6.34 (*d*, 2.0)	6.36 (*d*, 2.0)	6.38 (*s*)	6.40 (*d*, 2.0)
2′	–	7.50 (*d*, 2.0)	7.51 (*d*, 2.0)	7.89 (*d*, 8.5)	7.90 (*d*, 8.5)
3′	–	–	–	6.90 (*d*, 8.5)	6.90 (*d*, 8.5)
5′	–	6.88 (*d*, 8.5)	6.88 (*d*, 8.5)	6.90 (*d*, 8.5)	6.90 (*d*, 8.5)
6′	–	7.45 (*dd*, 8.5, 2.0)	7.46 (*dd*, 8.5, 2.0)	7.89 (*d*, 8.5)	7.90 (*d*, 8.5)
Sugar moiety					
(3)-*O*-Sugar					
H-1	5.77 (*d*, 8.0)	6.18 (*d*, 8.0)	6.19 (*d*, 8.0)	6.16 (*d*, 8.0)	6.17 (*d*, 8.0)
2	4.91 (*dd* 8.5, 8.0)	3.58 *	3.58 *	3.53 *	3.52 *
3	3.59 *	3.84 (*t*, 9.0)	3.85 (*t*, 9.0)	3.85 (*t*, 9.0)	3.84 (*t*, 9.0)
4	3.39 (*t*, 9.0)	3.34 (*t*, 9.0)	3.34 (*t*, 9.0)	3.32 (*t*, 9.0)	3.31 *
5	3.59 *	3.27 (*m*)	3.27 (*m*)	3.29 *	3.27 (*m*)
6	4.05 (*brd*, 9.5)	3.67 (*dd*, 12.0, 2.5)	3.67 (*dd*, 12.0, 2.0)	3.67 (*brd*, 12.0)	3.67 (*dd*, 12.0, 2.0)
6	3.67 *	3.51 (*dd*, 12.0, 5.5)	3.51 *	3.50 (*dd*, 12.0, 5.5)	3.49 (*dd*, 12.0, 5.5)
1′	4.20 (*d*, 8.0)	5.24 (*d*, 8.0)	5.25 (*d*, 8.0)	5.25 (*d*, 8.0)	5.24 (*d*, 8.0)
2′	2.99 (*dd*, 9.0, 8.0)	4.93 (*dd*, 9.0, 8.0)	4.93 (*dd*, 9.0, 8.0)	4.95 (*dd*, 9.0, 8.0)	4.94 (*dd*, 9.0, 8.0)
3′	3.14 (*t*, 9.0)	3.80 (*t*, 9.0)	3.80 (*t*, 9.0)	3.81 (*t*, 9.0)	3.79 (*t*, 9.0)
4′	3.08 *	3.53 *	3.54 *	3.53 *	3.52 *
5′	3.08 *	3.53 *	3.52 *	3.53 *	3.52 *
6′	3.67 *	3.93 (*brd*, 12.0)	3.93 (*brd*, 12.0)	3.91 *	3.93 (*dd*, 12.0, 2.0)
6′	3.45 *	3.78 (*dd*, 12.0, 4.0)	3.78 (*dd*, 12.0, 4.0)	3.78 (*dd*, 12.0, 4.0)	3.77 (*dd*, 12.0, 5.5)
7-*O*-Sugar					
H-1″	–	5.13 (*d*, 8.0)	5.10 (*d*, 8.0)	5.13 (*d*, 8.0)	5.10 (*d*, 8.0)
2″	–	3.57 (*dd*, 9.0, 8.0)	3.51 *	3.58 (*dd*, 9.0, 8.0)	3.50 *
3″	–	3.75 (*t*, 9.0)	3.58 *	3.75 (*t*, 9.0)	3.58 *
4″	–	3.69 (*t*, 9.0)	3.42 (t, 9.0)	3.69 (*t*, 9.0)	3.41 (*t*, 9.0)
5″	–	3.72 *	3.57 *	3.72 *	3.57 *
6″	–	3.98 (*dd*, 12.0, 2.2)	3.95 (*dd*, 12.0, 2.5)	3.99 (*brd*, 12.0)	3.94 (*dd*, 12.0, 2.0)
6″	–	3.93 (*brd*, 12.0)	3.74 (*dd*, 12.0, 6.0)	3.91 *	3.73 (*dd*, 12.0, 6.0)
H-1	–	4.48 (*d*, 8.0)	–	4.48 (*d*, 8.0)	–
2‴	–	3.29 (*dd*, 9.0, 8.0)	–	3.30 *	–
3‴	–	3.42 (*t*, 9.0)	–	3.43 (*t*, 9.0)	–
4‴	–	3.34 (*t*, 9.0)	–	3.35 (*t*, 9.0)	–
5‴	–	3.39 *	–	3.40 *	–
6‴	–	3.92 (*dd*, 12.0, 2.5)	–	3.91 *	–
6‴	–	3.70 *	–	3.70 *	–
Ester moiety					
H-*β*	6.49 (*d*, 16.0)	6.09 (*d*, 16.0)	6.09 (*d*, 16.0)	6.10 (*d*, 16.0)	6.10 (*d*, 16.0)
*γ*	7.55 (*d*, 16.0)	7.30 (*d*, 16.0)	7.30 (*d*, 16.0)	7.31 (*d*, 16.0)	7.31 (*d*, 16.0)
2,6	6.99 (*s*)	6.23 (*s*)	6.23 (*s*)	6.25 (*s*)	6.25 (*s*)
OMe	3.77 (*s*)× 2	3.59 (*s* ) × 2	3.59 (*s*) × 2	3.59 (*s*) × 2	3.60 (*s*) × 2
H-*β*′	6.44 (*d*, 16.0)	–	–	–	–
*γ*′	7.53 (*d*, 16.0)	–	–	–	–
2′,6′	6.96 (*s*)	–	–	–	–
OMe′	3.78 (*s*) × 2	–	–	–	–

^a^: Measured in DMSO-*d*_6_. ^b^: Measured in MeOH-*d*_4_. *: Overlapping with other signals.

**Table 3:** Effect of various compounds in the cytotoxicity assay.

Test Substance	Control	1	10	100
	(µM)			
	Viable Cell Rate (% of Control)			
**1**	100.0 ± 0.9	105.0 ± 1.6	101.4 ± 1.1	103.1 ± 0.7
**2**	100.0 ± 0.6	96.0 ± 0.9	94.9 ± 1.1	96.1 ± 1.0
**3**	100.0 ± 0.9	97.9 ± 0.4	98.9 ± 1.1	103.8 ± 1.1
**4**	100.0 ± 1.1	88.6 ± 0.8	93.5 ± 1.8	91.6 ± 0.7
**5**	100.0 ± 0.2	98.5 ± 2.5	102.0 ± 1.1	99.5 ± 0.8
Quercetin (**6**)	100.0 ± 0.6	102.0 ± 0.4	98.0 ± 1.1	76.6 ± 1.2 *
Kaempferol (**7**)	100.0 ± 1.2	105.7 ± 2.5	111.1 ± 1.7	66.6 ± 1.0 *
Sinapic acid (**8**)	100.0 ± 1.4	110.0 ± 1.4	107.0 ± 0.9	119.1 ± 2.4
Ferulic acid (**9**)	100.0 ± 0.6	102.4 ± 1.2	108.4 ± 1.3	111.5 ± 0.3
Caffeic acid (**10**)	100.0 ± 0.1	107.0 ± 1.7	107.8 ± 1.6	101.0 ± 2.4

Each value represents the mean ± S.E. for 3 determinations. * *p* < 0.05, significantly different from the control (0.1% DMSO).

**Table 4:** Effects of various compounds on glucose uptake by C2C12 skeletal muscle cells in the absence of insulin.

Test Compounds	Control	1	10	100
	(µM)			
	Uptake (% of Control)			
**1**	100.0 ± 2.8	128.4 ± 5.5 **	125.2 ± 3.2 ***	122.1 ± 1.4 ***
**2**	100.0 ± 2.8	130.9 ± 8.0 *	108.7 ± 4.8	88.3 ± 1.8 *
**3**	100.0 ± 3.6	130.7 ± 6.9 **	120.1 ± 1.9 **	100.8 ± 3.7
**4**	100.0 ± 3.6	110.4 ± 5.6	127.4 ± 5.0 **	145.5 ± 2.8 ***
**5**	100.0 ± 3.7	116.8 ± 3.5 *	114.9 ± 2.5 *	103.0 ± 4.7
Quercetin (**6**)	100.0 ± 3.7	104.1 ± 3.5	102.3 ± 2.8	25.5 ± 0.7 ***
Kaempferol (**7**)	100.0 ± 4.4	135.0 ± 2.4 ***	144.5 ± 9.5 **	39.8 ± 1.4 ***
Sinapic acid (**8**)	100.0 ± 4.4	135.8 ± 6.8 **	129.7 ± 4.9 **	127.3 ± 3.7 **
Ferulic acid (**9**)	100.0 ± 3.7	105.6 ± 2.4	120.5 ± 2.8 **	116.2 ± 1.5 **
Caffeic acid (**10**)	100.0 ± 3.7	129.9 ± 11.7	111.9 ± 5.0	108.6 ± 7.1

After treatment with various compounds at the indicated concentration in the absence of insulin, glucose uptake was measured using the Promega Glucose Uptake-Glo™ Assay kit. Each value represents the mean ± S.E. for 5 determinations. *** *p* < 0.001, ** *p* < 0.01, * *p* < 0.05 represent significant differences from the control (0.1% DMSO).

**Table 5:** Effects of various compounds on glucose uptake by C2C12 skeletal muscle cells in the presence of insulin.

Test Compounds	Control	1	10	100
	(µM)			
	Uptake (% of Control)			
**1**	100.0 ± 8.4	96.3 ± 4.0	92.1 ± 2.9	81.8 ± 1.8
**2**	100.0 ± 8.4	84.4 ± 1.9	82.2 ± 3.9	83.8 ± 5.2
**3**	100.0 ± 3.1	110.6 ± 2.6 *	120.5 ± 7.4	81.9 ± 4.6 *
**4**	100.0 ± 3.1	74.2 ± 2.9 ***	90.5 ± 1.7	123.1 ± 9.4 *
**5**	100.0 ± 3.5	114.5 ± 5.4	103.7 ± 5.7	81.1 ± 4.4 *
Quercetin (**6**)	100.0 ± 3.5	72.9 ± 5.5 **	76.8 ± 3.7 **	20.1 ± 1.8 ***
Kaempferol (**7**)	100.0 ± 4.6	148.4 ± 6.8 ***	132.7 ± 2.3 ***	31.7 ± 1.0 ***
Sinapic acid (**8**)	100.0 ± 4.6	116.5 ± 1.5 *	119.3 ± 2.7 *	123.0 ± 3.1 **
Ferulic acid (**9**)	100.0 ± 8.6	97.0 ± 2.0	118.7 ± 7.3	94.9 ± 2.2
Caffeic acid (**10**)	100.0 ± 8.6	94.3 ± 1.9	101.7 ± 5.6	99.5 ± 0.7

After treatment with various compounds at the indicated concentration in the presence of insulin, glucose uptake was measured using the Promega Glucose Uptake-Glo™ Assay kit. Each value represents the mean ± S.E. for 5 determinations. *** *p* < 0.001, ** *p* < 0.01, * *p* < 0.05 represent significant differences from the control (0.1% DMSO).

## Data Availability

The data presented in this study are available from the corresponding author upon reasonable request.

## Supplementary Materials

The following supporting information can be downloaded at https://www.mdpi.com/article/10.3390/biom14101246/s1. Chart S1: ^1^H and ^13^C NMR spectra of compounds **1**–**4** and **5**.
