# Supplementary material for: Identification of Sinapic Acid Derivatives from Petit Vert Leaves and Their Effects on Glucose Uptake in C2C12 Murine Myoblasts
Source: Biomolecules. 2024 Oct 1;14(10):1246. doi: 10.3390/biom14101246 (PMC11505672; doi:10.3390/biom14101246)

Chart S1. ^{1}H and ^{13}C NMR spectra of compounds 1–4, and 5

^{1}H and ^{13}C NMR spectra of compound 1

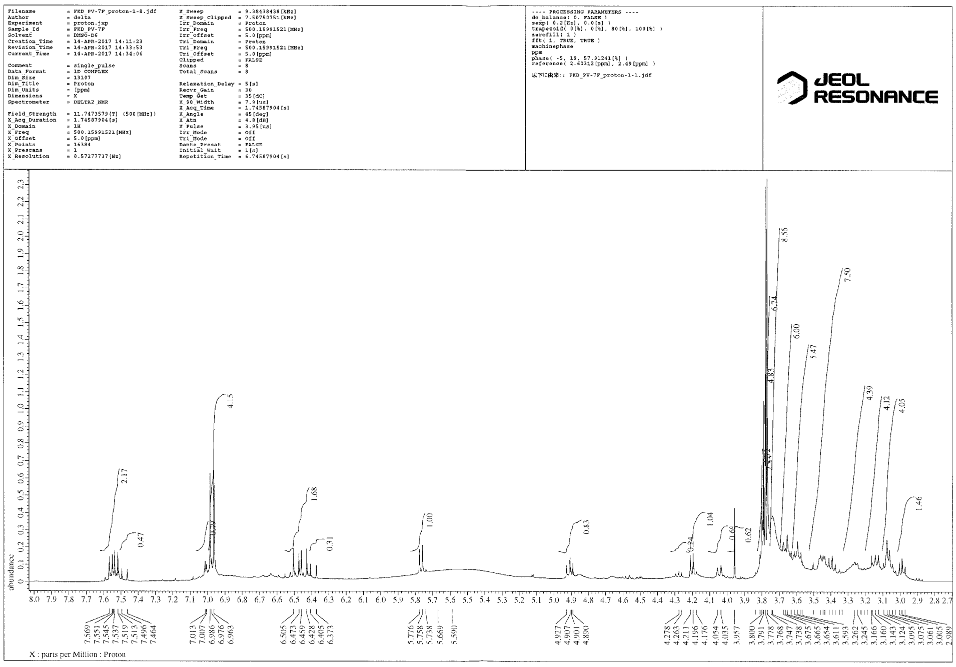

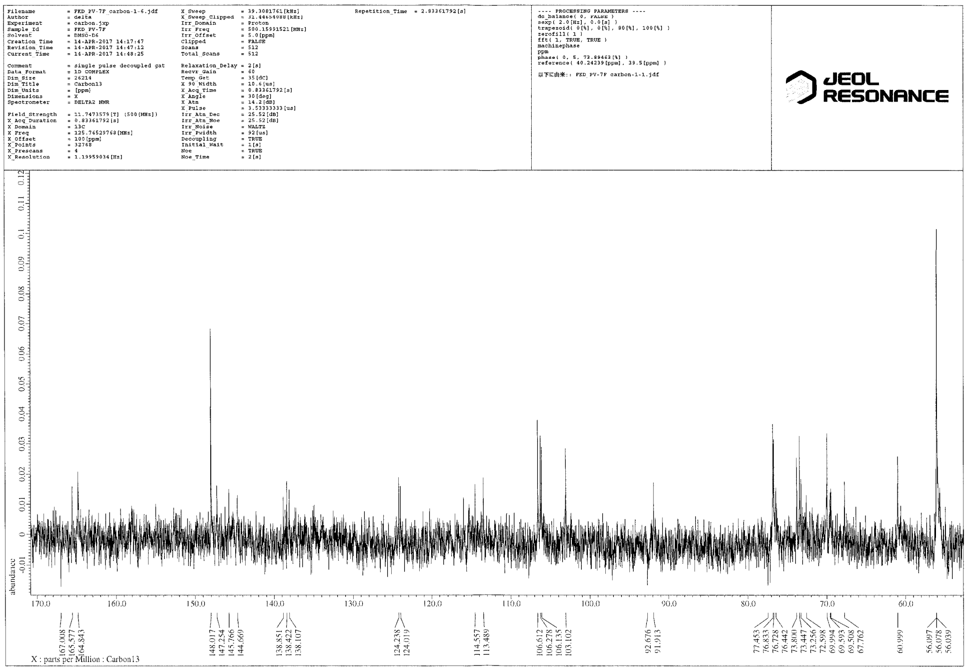

^{1}H and ^{13}C NMR spectra of compound **2**

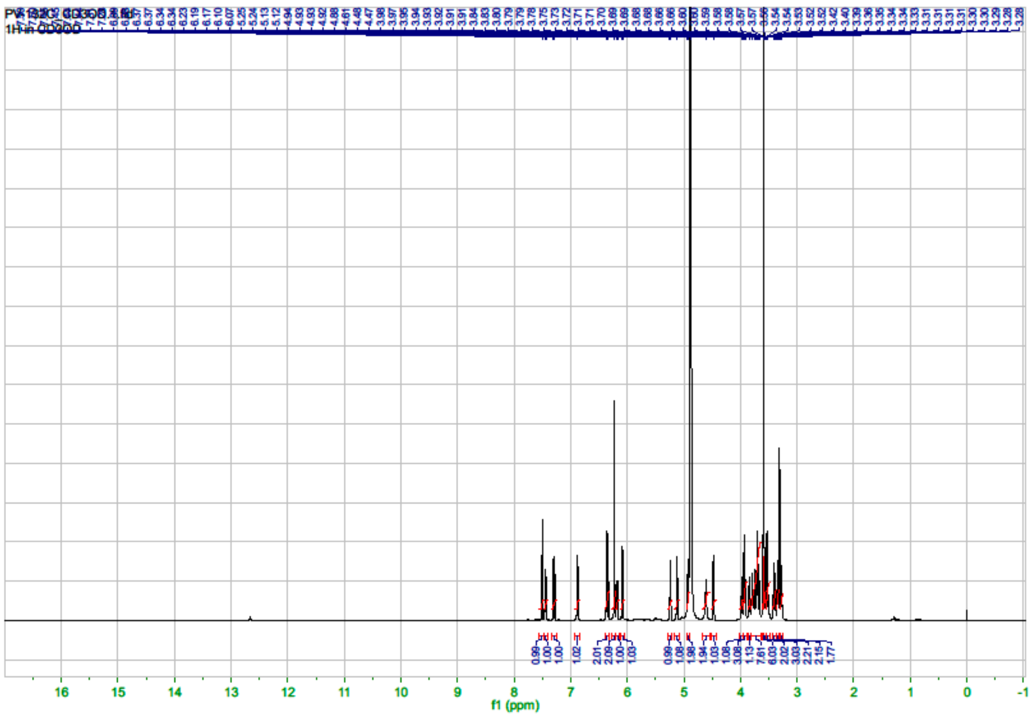

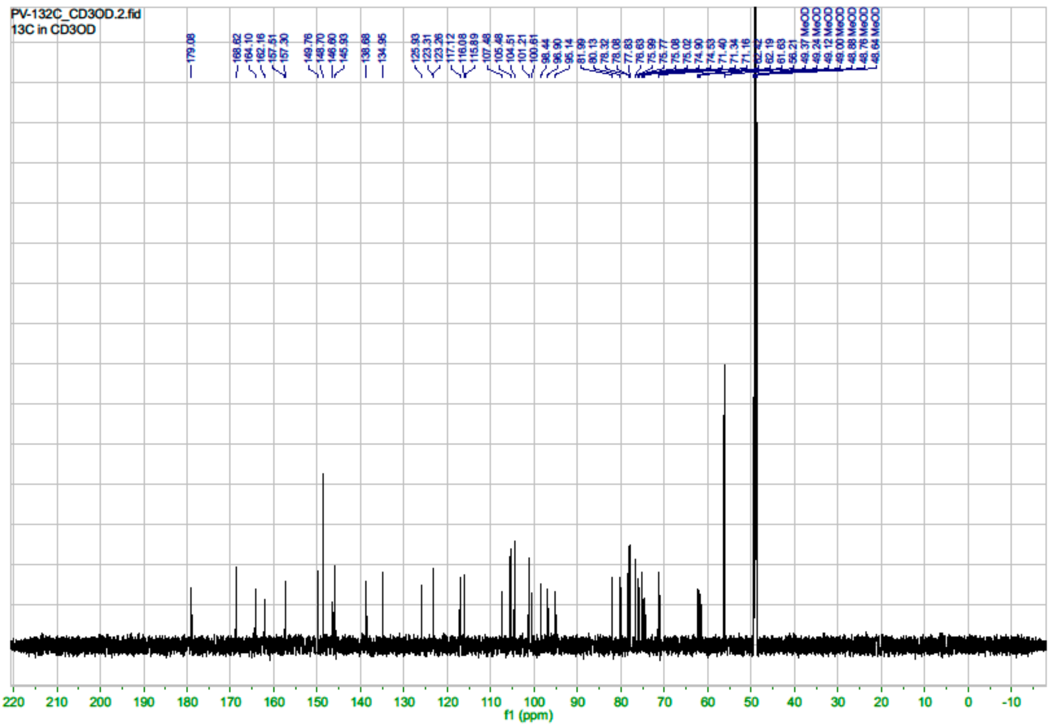

^{1}H and ^{13}C NMR spectra of compound 3

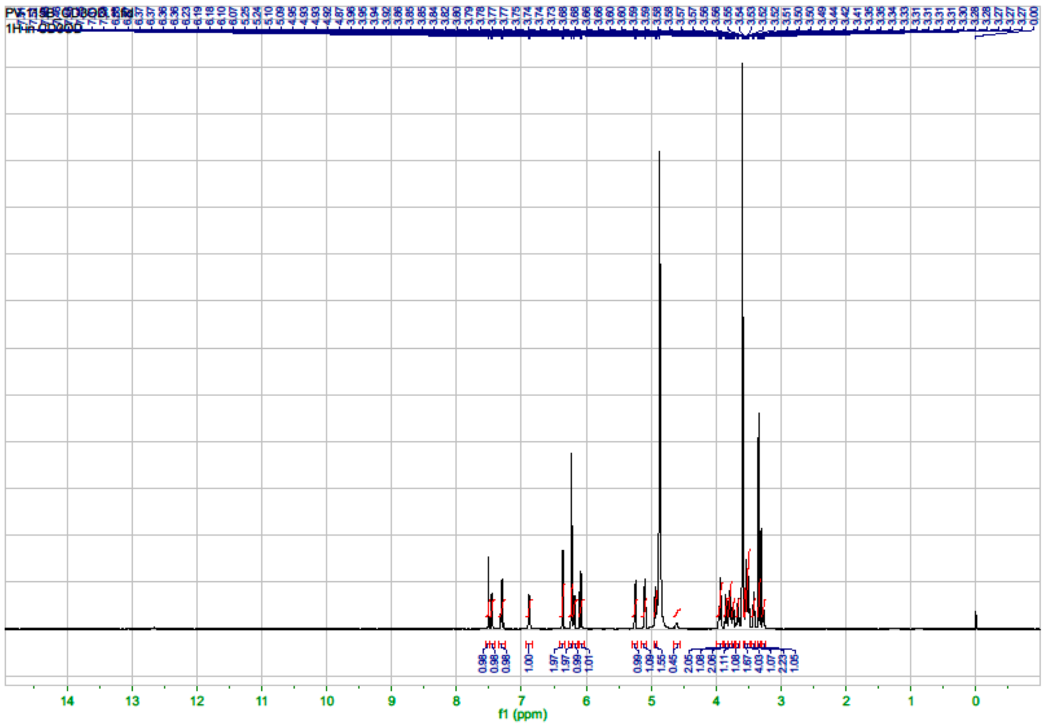

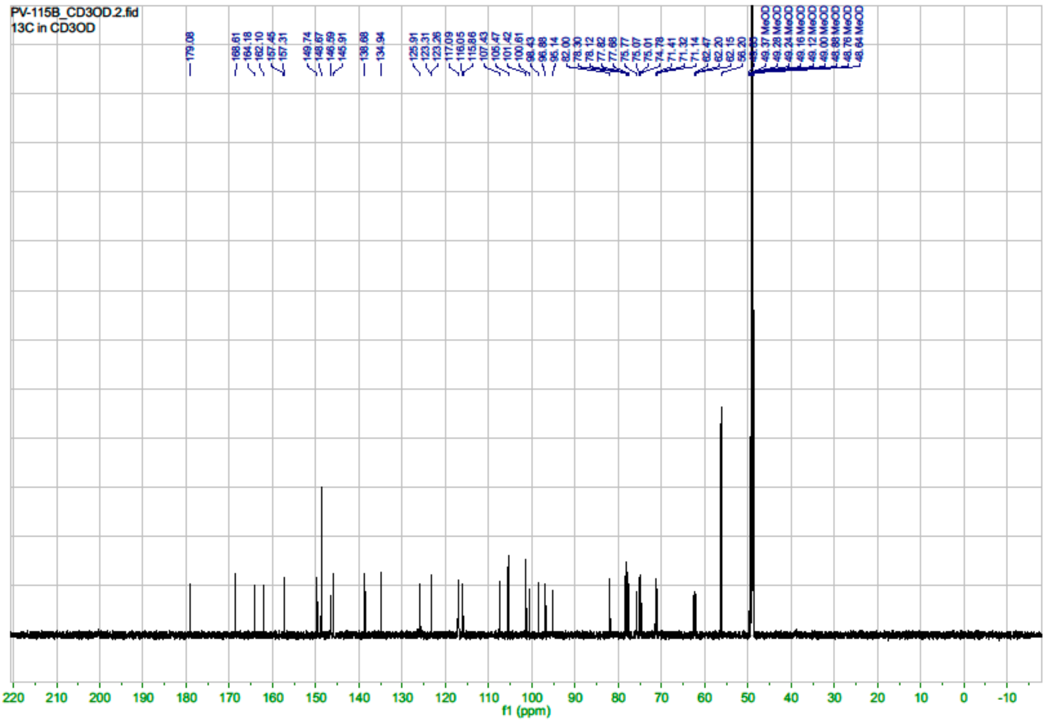

¹H and ¹³C NMR spectra of compound 4

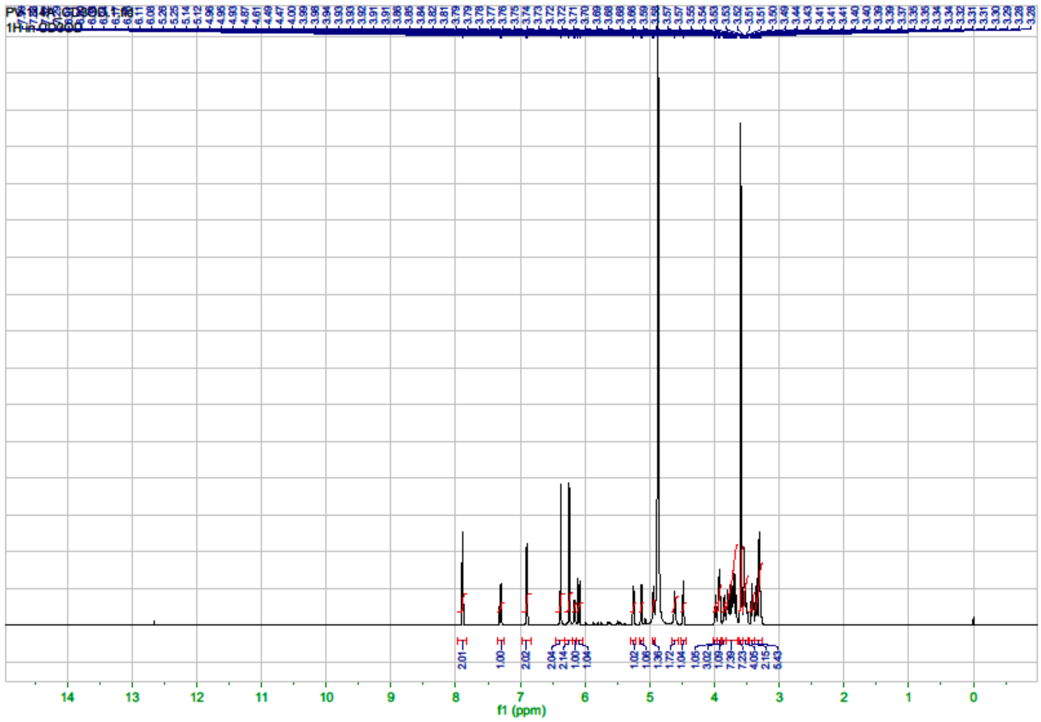

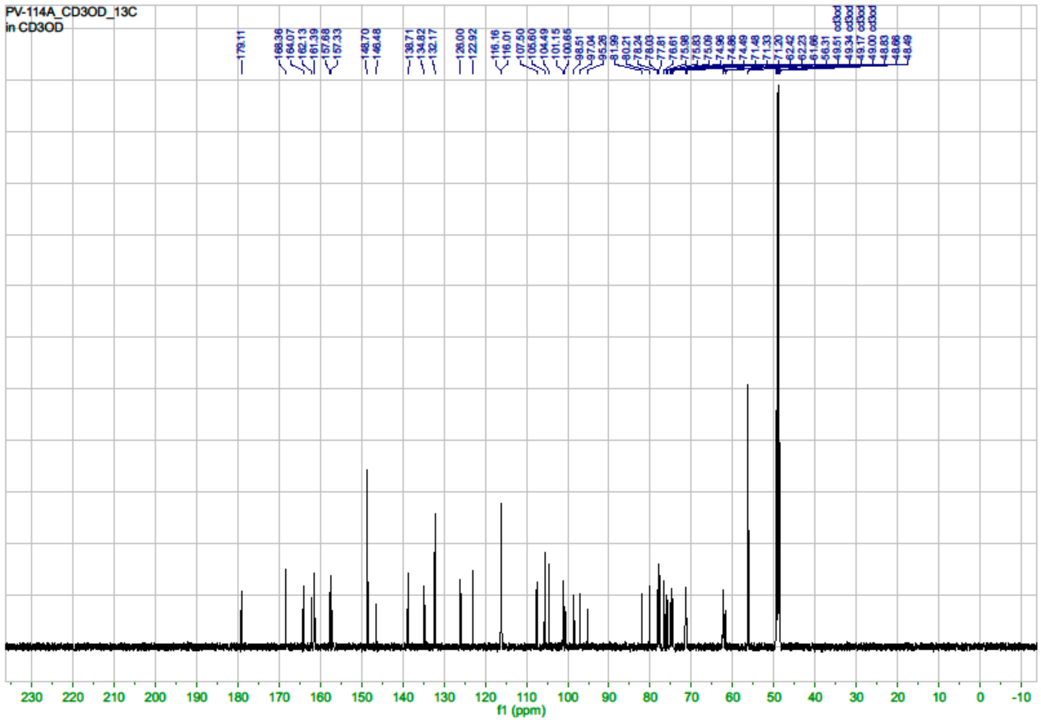

^1H and ^{13}C NMR spectra of compound **5**

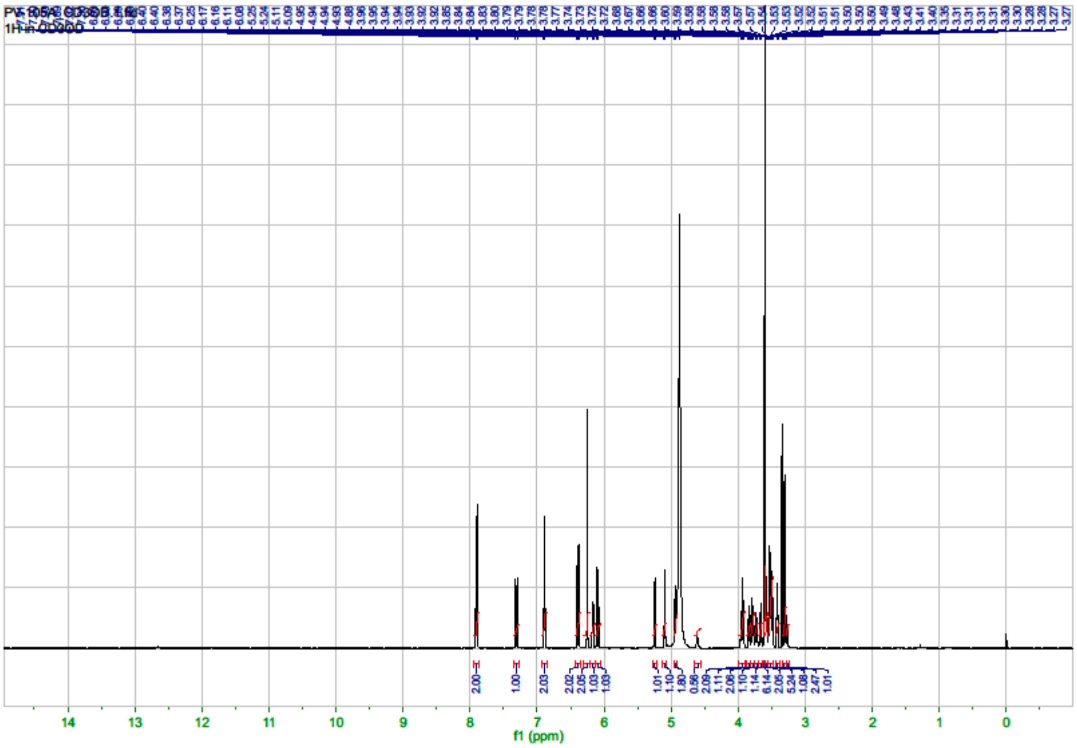

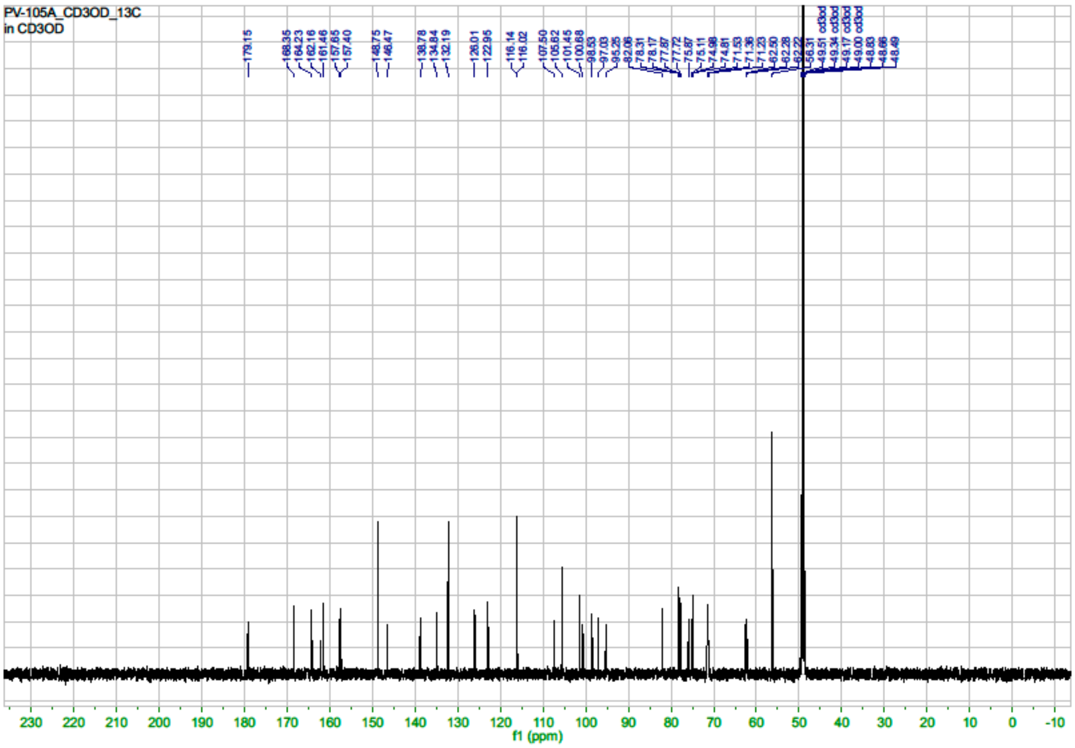

Supplement: Supplementary file 1 [file biomolecules-14-01246-s001.zip › biomolecules-3193047-supplementary.pdf]
